# Graphitic Carbon Nitride Doped Copper–Manganese Alloy as High–Performance Electrode Material in Supercapacitor for Energy Storage

**DOI:** 10.3390/nano10010002

**Published:** 2019-12-18

**Authors:** Samarjeet Singh Siwal, Qibo Zhang, Changbin Sun, Vijay Kumar Thakur

**Affiliations:** 1 Key Laboratory of Ionic Liquids Metallurgy, Faculty of Metallurgical and Energy Engineering, Kunming University of Science and Technology, Kunming 650093, China; changbin_sun@163.com; 2 State Key Laboratory of Complex Nonferrous Metal Resources Cleaning Utilization in Yunnan Province, Kunming 650093, China; 3 Enhanced Composites and Structures Center, School of Aerospace, Transport and Manufacturing, Cranfield University, Bedfordshire MK43 0AL, UK; 4 Department of Mechanical Engineering, School of Engineering, Shiv Nadar University, Uttar Pradesh 201314, India

**Keywords:** copper-manganese alloy, energy storage, supercapacitor, graphitic carbon nitride

## Abstract

Here, we report the synthesis of copper–manganese alloy (CuMnO_2_) using graphitic carbon nitride (gCN) as a novel support material. The successful formation of CuMnO_2_-gCN was confirmed through spectroscopic, optical, and other characterization techniques. We have applied this catalyst as the energy storage material in the alkaline media and it has shown good catalytic behavior in supercapacitor applications. The CuMnO_2_-gCN demonstrates outstanding electrocapacitive performance, having high capacitance (817.85 A·g^−1^) and well-cycling stability (1000 cycles) when used as a working electrode material for supercapacitor applications. For comparison, we have also used the gCN and Cu_2_O-gCN for supercapacitor applications. This study proposes a simple path for the extensive construction of self-attaining double metal alloy with control size and uniformity in high-performance energy-storing materials.

## 1. Introduction

Currently, there is a great thrust on the usage of two-dimensional (2D) graphitic carbon nanomaterials for energy storage owing to their novel electronic and other characteristics [1,2,3]. The faradic response within 2D graphitic carbon also advances their electrochemical energy storing activity and, for this, the overview of heteroatoms, for example, nitrogen, must be confirmed as a capable method [4,5,6,7]. Owing to the enhanced feasting of universal energy sources, significant efforts have been dedicated to the advancement of feasible energy renovation/storage strategies [8,9,10,11]. Indeed, the growth of environmentally-friendly energy renovation/storing strategies has developed significant worries that require sufficient explanation to preserve the feasibility of our atmosphere. In this direction, supercapacitor denotes a novel type of energy storing strategy among economical capacitors, as well as for chargeable batteries, which carries high power density along with an extensive lifecycle [12,13]. However, its small energy density (<10 Wh·kg^−1^) significantly limits its real-world use [14].

The notable mechanical and chemical characteristics including thermal flexibility of carbon nitrides linked by their surface and intralayer chemical reactivity have led to the opportunities for elaborating carbon nitride substances for catalysis uses, either intrinsically or while adorned by metal/metal oxide nanoparticles (NPs). Graphitic carbon nitride (gCN) substances have been revealed to act as metal-free heterogeneous reactants, relying on the inherent Brønsted acid and Lewis base functionalities that give catalytically active positions [15,16]. Among several allotropes of carbon nitride, gCN is the most studied because it has semiconductor behavior with a bandgap of 2.7 eV [17]. Thin films of gCN shaped throughs-triazine/heptazine have been compared with hydrogen bonding to form a structure parallel to the graphite [18]. gCN holds outstanding characteristics, for example, long thermal steadiness able near 600 °C in air, defiance towards numerous chemicals, high N_2_ contented, ecologically benevolent properties, and so on. While unpackaged gCN shows more theoretic N_2_ contented (C/N proportion is 0.75), it is exciting for manufacturing gCN by perfect stoichiometry via physical/chemical approaches as the development of the C–N chain is thermodynamically unfavorable, then polycondensation of the pioneer’s issues N_2_ particle in its place of establishing the C–N bond [14]. Furthermore, the intrinsic small surface area and the nonporous behavior of bulk gCN have restricted their use within electrocatalysis, energy-storing, chemisorption, and so on [19]. Towards mitigation of these issues, Vinu et al. [20] adopted a rigid pattern process that effectively produced permeable, more surface area of gCN. Consequently, owing to the achieved high surface area, permeable gCN was exposed to show virtuous catalytic movement, power, and gas storing ability [21]. By the way, transition metal sulfides (TMSs), particularly, manganese sulfides (MnS), have been well-thought-out as probable substantial materials aimed at supercapacitor (SC) applications [22]. gCN-doped MnS has accomplished characteristics, for example, higher theoretic specific capacitance (463.32 F·g^−1^), high durability, low price, profuse, and ecological flora [23]. Mesoporous gCN substances exhibit the maximum specific capacitance around 286 F·g^−1^ and a current density of 0.75 A·g^−1^ [24]. Further, Xiaoyang et al. [25] have reported their study on NiMoO_4_ films that were grown on gCN employing a facile chemical precipitation protocol that shows a significant specific capacitance of 1275 F g^−^¹ on 0.25 A·g^−^¹ owing to the interconnected composition as well as appearance of N with the inclusion of gCN.

Numerous nanostructured resources comprising metal alloys, metal oxides, and metal hydroxides [26,27,28,29] are being extensively employed within electrochemical SCs owing to their lower price, natural profusion, and outstanding charge storing aptitude [30]. The use of transition metal oxides (e.g., Co_3_O_4_, MnO_2_, and CuO) is an economical approach to advance catalytic converters with improved surface area, permeability, activity, and durability to name a few [31]. In our present work, we have synthesized gCN doped copper (I) oxide nanoparticle and manganese–copper alloys with exceptional surface area and homogenous distribution of CuMnO_2_ for exposing more active sites. The different analyses and catalytic performances show the better utility of this material for SC applications. From the different characterizations, the formation of Cu_2_O-gCN and CuMnO_2_-gCN with the porous structure was confirmed. For comparison, we have studied the gCN and Cu_2_O-gCN nanoparticles and found a massive improvement in the catalytic performance after introducing the Mn to the system. This is the facile route to synthesize the energy storage materials at room temperature without any specific equipment with excellent stability and charge storage capability. The CuMnO_2_-gCN manifests excellent electrocapacitive performance with high capacitance (817.85 A·g^−1^) and well-cycling stability (1000 cycles) used as working electrode material for supercapacitor applications.

## 2. Materials and Methods

### 2.1. Wide-Ranging Process for the Synthesis of gCN, Cu_2_O-gCN, and CuMnO_2_-gCN

In representative operation, 20 g of urea was retained under a closed porcelain vessel at 60 °C following atmospheric condition for 4 h. Afterward, this precursor was shifted in a muffle kiln for 4 h at 450 °C [32,33,34]. The pale-yellow-stock material gCN was rinsed numerous times using deionized water to eliminate the remaining alkaline sorts over the specimen exterior and again dried at 60 °C for 24 h. In the subsequent step, the collected material was dispersed in 100 mL of water in a 250 mL conical flask. The Cu_2_O-gCN was amalgamated using a single-step borohydride reduction method at room temperature. An aqueous suspension of copper sulphate pentahydrate (10^−1^ mol·dm^−3^) was annexed dropwise (5 wt % loading of Cu) to the round-bottom container. Subsequently, 5 mL of NaBH_4_ (10^−2^ mol·dm^−3^) solution was added gradually, aiming at the reduction of the Cu salt. Lastly, the substance was penetrated, rinsed through water, and then dried. Similarly, CuMnO_2_ (5.0 mol % of Cu and Mn loading) was also synthesized employing the lineages of CuSO_4_·5H_2_O and KMnO_4_ powder. Lastly, the material was separated, rinsed with water numerous times, and dried at 70 °C for 24 h following vacuum condition.

### 2.2. Electrode Modification

The anode electrodes were fabricated through drop-casting gCN, Cu_2_O-gCN, and CuMnO_2_-gCN over a glassy carbon electrode (GCE). Before deposition, the GCE was swept via sonicating in ethanol around 2 min then washing successively by acetone along with deionized water. During drop-casting, the substance suspension was fabricated through scattering 2 mg of gCN, Cu_2_O-gCN and CuMnO_2_-gCN powder individually within 1 mL of 4:1 *v*/*v* ethyl acetate/5% Nafion through sonication after drop-casted upon a GCE by a catalyst packing of 0.2 mg·cm^−2^ then leave at room temperature for 2 h. The coating width was examined by changing the deposition period.

### 2.3. Electrochemical Experiments

Electrochemical study was carried out using a Shanghai Chenhua 760 E potentiostat within a single-cell three-electrode system in a 0.5 mol·dm^−3^ KOH solution. The electrochemical containers were cleaned by aqua regia and then millipore water before further analyses. Glassy carbon, Pt column, and Hg/HgO^+^ (3.0 mol·dm^−3^ KOH) were adopted as the working, counter, and reference electrodes, respectively. The charging–discharging of the catalyst was measured utilizing chronopotentiometry at various current densities. In addition, electrochemical impedance spectroscopy (EIS) analyses were conducted in the frequency range from 3 MHz to 10 Hz.

Specific capacitance (*C_S_*), power density (*P*), plus energy density (*E*) are received from the galvanostatic discharge arcs conferred by the subsequent equations [35]:

(1) $$C_{s}\;=\;\frac{I\Delta t}{m\Delta V}\left(F\cdot g^{-1}\right)$$

(2) $$E\;=\;\frac{0.5C_{S}\Delta V^{2}}{3.6}\left(Wh\cdot kg^{-1}\right),$$

(3) $$P\;=\;\frac{3600E}{\Delta t}\left(F\cdot g^{-1}\right).$$

Here, *I* represents the discharge current, Δ*t* displays the complete discharge period, and Δ*V* is the potential period of complete discharge.

### 2.4. Characterization

The X-ray diffraction (XRD) pattern was carried out on a Rigaku X-ray diffractometer (MinifexII Desktop) with Cu *Kα* radiation (Rigaku Corporation, Tokyo, Japan). Field-emission scanning electron microscopy (FESEM; Nova 400 Nano-SEM, Nova High-Technologies Corporation, Abingdon, UK) was carried out at an expedited voltage of 15 kV. Transmission electron microscopy (TEM) analyses were conducted at 200 kV applying Tecnai G2 TF30 (JEOL, Tokyo, Japan) transmission microscopy. X-ray photoelectron spectroscopy (XPS) data were collected by a PHI 550 (Thermo Fisher Scientific, Waltham, MA, USA) spectrometer employing a monochromatic Al–*Kα* (1486.6 eV) radiation origin and a hemispheric detector including an energy resolution of 0.1 eV. Materials surface area and pore size spreading of the specimens were estimated by N_2_ adsorption–desorption depending upon the Brauner–Emmet–Teller (BET) plus Barrett–Joyner–Halenda (BJH) (Quantachrome Instrument, Boynton Beach, FL, USA) method (Belsorp-BELMAX).

## 3. Results and Discussion

### 3.1. Morphological and Structural Analysis

**Figure 1:**
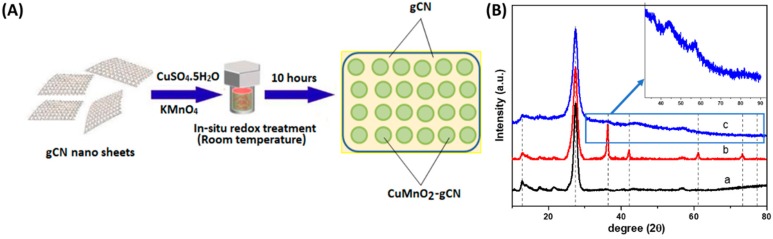
(**A**) Schematic representation of the catalyst synthesis. (**B**) X-ray diffraction (XRD) pattern of (**a**) graphitic carbon nitride (gCN), (**b**) Cu_2_O-gCN, and (**c**) CuMnO_2_-gCN.

The schematic representation of the catalyst synthesis is shown in Figure 1A. The characteristics of a crystallographic assembly of gCN nanosheets, Cu_2_O-gCN, and CuMnO_2_-gCN nanohybrids with the varied quantity of gCN are shown in Figure 1B. The gCN nanosheets generally showed a very weak peak on 13.1° (100), then an additional separate peak on 27.5° indexed towards (002) planes of hexagonal graphitic carbon assembly, assigned towards the inter-film filling and interplanar assembling peaks of the aromatic structure, correspondingly [36]. Meanwhile, the peaks at 13.1°, 27.7°, 36.4°, 42.3°, 61.3°, 73.5° and 77.3° are indexed as (100), (002), (111), (200), (220), (311), and (222), respectively, and the characteristic diffractions of the hydrotalcite crystalline assembly of Cu_2_O-gCN nanohybrids [37,38]. The peak intensity on 27.5° elevated deprived of upsetting the location of the peak of Cu_2_O-gCN by mounting the weight of gCN nanofilms upon Cu. It intended that the crystal development of gCN nanofilms did not hinder the crystal growing of Cu_2_O-gCN. While we introduced the Mn to the above system, the nature of the material changed from the crystalline to amorphous and the peaks corresponding to the Cu_2_O gone in the final CuMnO_2_-gCN product. The High-resolution Transmission Electron Microscopy (HRTEM) and fast Fourier transform (FFT) studies also confirm the amorphous nature of the CuMnO_2_-gCN material. Hereafter, the recommended two dissimilar paths for the development of CuMnO_2_ are as follows [39]:

(a) First step:
  (4) $$\frac{1}{\left(3-x\right)}{Cu}_{x}{Mn}_{3-x}O_{4}\;+\;\frac{\left(3-2x\right)}{3-x}CuO\;\to \;\frac{1}{2\left(3-x\right)}O_{2}\;+\;CuMnO_{2}\left(x=1\right)$$

(b) Second step:
  (5) $$2CuO\;\to \;Cu_{2}O\;+\;\frac{1}{2}O_{2},$$
  (6) $$Mn_{2}O_{3}\;+\;Cu_{2}O$$

Equations (5) and (6) give the final reaction:

(7) $$2CuO\;+\;Mn_{2}O_{3}\;\to \;2CuMnO_{2}\;+\;\frac{1}{2}O_{2}\;$$

**Figure 2:**
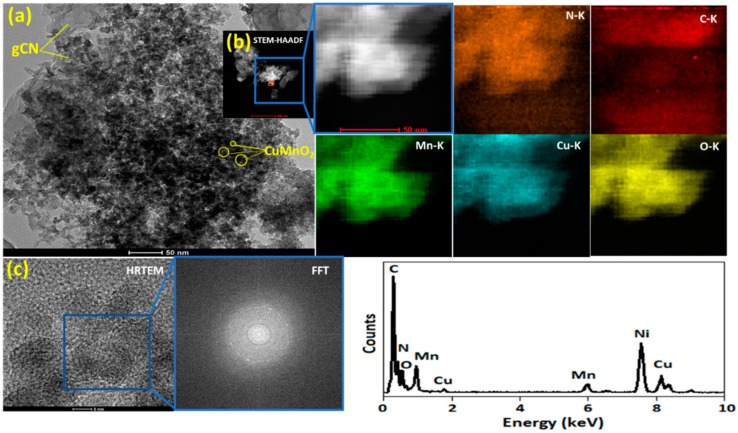
(**a**) Transmission electron microscopy (TEM), (**b**) STEM-HAADF images of CuMnO_2_-gCN and corresponding EDX elemental mapping of the selected area in (**b**,**c**) HRTEM image of CuMnO_2_-gCN and corresponding fast Fourier transform (FFT) pattern of the selected area.

The morphology and corresponding energy-dispersive X-ray spectrum (EDX) element mapping of gCN sheets, which are employed as a precursor to construct Cu_2_O-gCN compounds, is shown in Figure S1. The gCN films can be identified obviously on the exterior of the sphere-shaped aggregates, which specifies the development of Cu_2_O-gCN composites (Figure S2). As reported, the formation of Cu_2_O-gCN aggregates is an outcome of the robust attraction among the MO_x_ and the abundant active groups of gCN [40]. The intimate contact between the gCN sheet and Cu_2_O microspheres was further confirmed by TEM, Scanning transmission electron microscope-High-angle annular dark-field (STEM-HAADF), and HRTEM images, as shown in Figure S3a–c. The TEM image (Figure 2a) of the CuMnO_2_-gCN complex displays that nanoparticles are perceived with a large size spreading, changing from 8 to 15 nm, on the gCN doped composite. Furthermore, the HAADF-STEM and its corresponding Energy-dispersive X-ray spectroscopy (EDS) element mapping images (Figure 2b) indicate the homogeneous distribution of N, C, O, Mn, and the doped Cu atoms in the whole materials [41]. The HRTEM images (Figure 2c) reveal no clear lattice fringes in the CuMnO_2_-gCN, emphasizing their amorphous structure, which is consistent with the results of the corresponding fast Fourier transform (FFT) pattern and the above XRD analysis.

**Figure 3:**
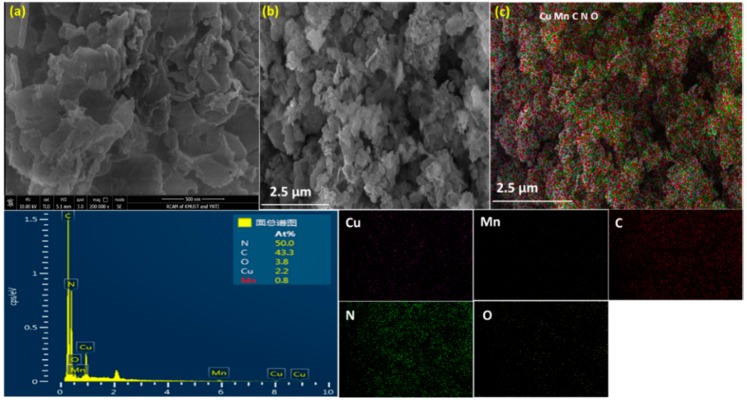
(**a**,**b**) Scanning electron microscopy (SEM) images and (**c**) corresponding EDS elemental mapping of CuMnO_2_-gCN.

The SEM images for the CuMnO_2_-gCN material conferred the appearance of gCN nanofilms and CuMnO_2_ nanoplates dispersed at the exterior of gCN by compact bits of comparable sizes and frames; the bit size is 0.5–2 µm (Figure 3a,b). As shown in Figure 3b, the docility of gCN observed from some points that are not incorporated through the nanoplates. The nanoplates exhibit comparable pattern and width essentially revealed in Figure 3a, describing that the addition of gCN does not influence the completion of CuMnO_2_ nanoplates. The disclosed compound of gCN nanofilms and CuMnO_2_ nanoplates could improve the surface area associated with Cu_2_O-gCN nanoplates (Figure S2). The EDS investigation (Figure 3c) explains that the resulting oxide comprises Cu, Mn, C, N, and O near stoichiometric proportions, recommending that the manufactured nanostructures are composed of absolute crednerite CuMnO_2_-gCN.

**Figure 4:**
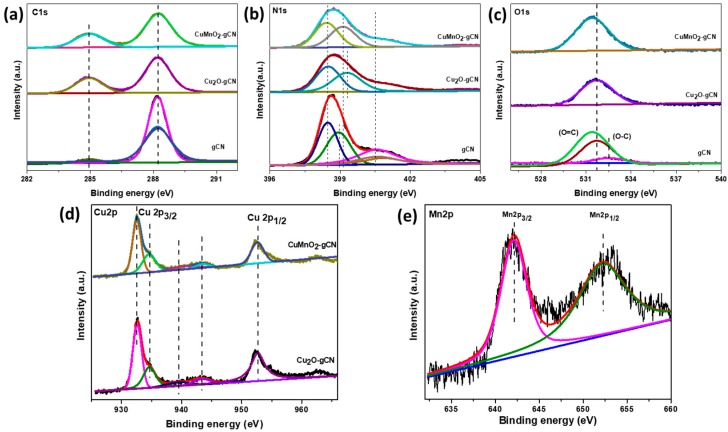
High-resolution X-ray photoelectron spectroscopy (XPS) spectra of (**a**) C1s, (**b**) N1s, (c) O1s, (**d**) Cu 2p, and (**e**) Mn 2p for gCN, Cu_2_O-gCN, and CuMnO_2_-gCN.

The introduction of carbon atoms into the composition of CN was additionally investigated by X-ray photoelectron spectroscopy (XPS). XPS core level spectra recommend that the bonding arrangement among carbon and N_2_ atoms toward the existing specimen is next to that of the gCN construction [42]. The C1s’ high-resolution XPS spectra (Figure 4a) of gCN display two main peaks at 284.9 eV and 288.2 eV, which might be attributed to defect-encompassing sp^2^-hybridized carbon particles [43,44]. The N1s’ high-resolution spectrum (Figure 4b) is deconvoluted within three peaks including binding energies about 400.9, 399.7, and 398.6 eV, which are associated with C–N–H, N–(C)_3_, and C–N=C components, correspondingly [45,46]. Furthermore, the close-fitting O1s’ spectra (Figure 4c) is categorized through three bands: two on 532.3 and 531.3 eV, owing to the fascination of oxygen and water particles upon the compound exterior, and then one at 530.4 eV, which resembles the O^2−^ band by Cu and Mn [47].

The XPS results disclose the existence of Cu_2_O nanocrystals, as revealed via the Cu 2p_3/2_ peaks in Figure 4d. Cu^+^ has a solitary peak at 932.2 eV with a full width half maximum (fwhm) of (1.64 ± 0.2 eV) [48,49] and shakeup satellites at ~939.35 and ~943.36 eV at higher binding energies. The Cu 2p_3/2_ aimed at the Cu_2_O-gCN and CuMnO_2_ composites were described at ~932.6 eV and~932.5 eV, correspondingly [50]. Inside the Cu spectra, two main peaks on ~932.6 eV (Cu 2p_3/2_) and then ~952.8 eV (Cu 2p_1/2_) by a splitting energy of 20.2 eV verify the monovalent state of Cu ion [51,52]. The XPS study spectra of the CuMnO_2_-gCN (Figure S4) approve the presence of C, Mn, Cu, N, and O. Figure 4e displays the high-resolution XPS spectrum of Mn 2p for Cu_2_O-gCN and CuMnO_2_-gCN. Within the Mn spectra, intense peaks at 642.1 eV (Mn 2p_3/2_) and 653.5 eV (Mn 2p_1/2_) by a splitting energy of 11.4 eV recognize the Mn^3+^ valance in the nanocomposite [41,53]. Two major peaks accredited towards Mn 2p_3/2_ and Mn 2p_1/2_ may be detected in mesoporous MnO_2_. After a peak-fitting deconvolution, the Mn 2p_3/2_ of mesoporous MnO_2_ can be defined one peak with Mn^4+^ ~ 642.1, and the conforming peaks of mesoporous MnO_2_ in Mn 2p_1/2_ can also be defined as one peak with Mn^4+^ ~ 653.5, demonstrating that Mn^4+^ is the key valence state of Mn in MnO_2_ [54].

**Figure 5:**
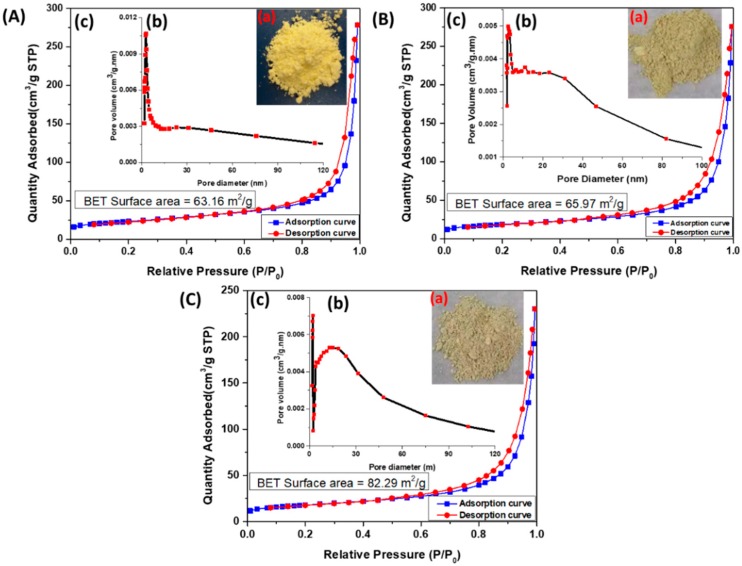
(**A**–**C**) The optical images (**a**,**b**) pore size distribution, and (**c**) nitrogen adsorption and desorption isotherms of gCN, Cu_2_O-gCN, and CuMnO_2_-gCN, respectively. BET, Brauner–Emmet–Teller.

Figure 5 shows the nitrogen adsorption and desorption isotherm for all the samples, gCN (A), Cu_2_O-gCN (B), and CuMnO_2_-gCN (C). These ingredients showed type IV isotherm through the hysteresis loop, which is an asset of the mesoporous substances [55]. The BET surface area experiments approve the augmented surface area of CuMnO_2_-gCN (82.29 m^2^·g^−1^) compared with Cu_2_O-gCN (65.95 m^2^·g^−1^) and gCN (63.16 m^2^·g^−1^), owing to the combination of Mn in the electrode material structure and the development of subordinate pores between gCN and CuMnO_2_. The improvement of surface area may deliver a higher amount of active positions, anticipated electrical linking for the profligate rate of a redox reaction, including charge transfer that could efficiently improve the energy storing capability. This remark is in acceptable agreement through the result of BJH pore size delivery (inset of Figure 5A–C), at which the maximum size of the CuMnO_2_-gCN nanocomposite is 12.5 nm, which can originate from the space among the CuMnO_2_ nanoplates plus gCN in addition to the nanosheets themselves. The isotherm was adapted to investigate the pore size distribution of the material by applying the Barrett-Joyner-Halenda (BJH) methods. The calculated BJH adsorption cumulative sizes of pores of the synthesized CuMnO_2_-gCN with a wide distribution of the pore radius of the material were noticed in Figure 5C along with the pore volume of 0.429886 cm^3^·g^−1^.

### 3.2. Electrochemical Studies

**Figure 6:**
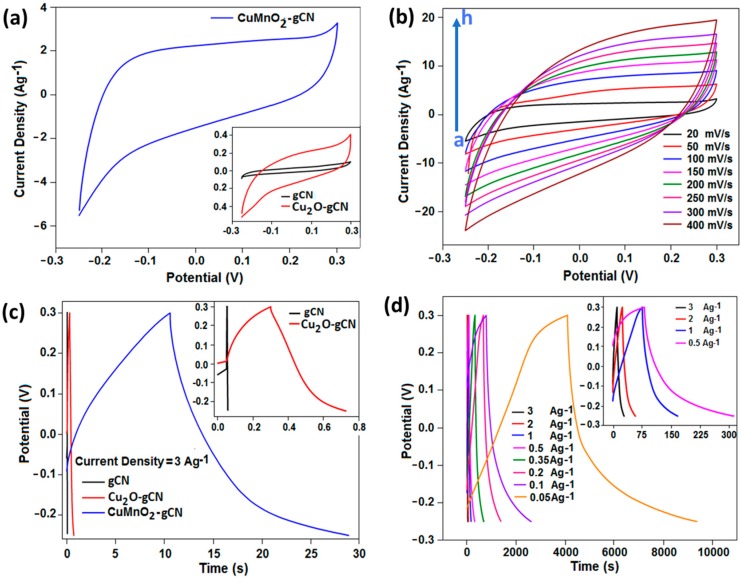
(**a**) Cyclic voltammetry (CV) curves of CuMnO_2_-gCN and inset figure for gCN and Cu_2_O-gCN study at scan rate of 20 mV·s^−1^ in 0.5 mol·dm^−3^ KOH. (**b**) The scan rate study of CuMnO_2_-gCN. (**c**) Galvanostatic charge–discharge (GCD) curves of gCN, Cu_2_O-gCN, and CuMnO_2_-gCN (the current density is 3 A·g^−1^). (**d**) GCD studies at diverse current densities of CuMnO_2_-gCN (whole study carried out in 0.5 mol·dm^−3^ KOH).

In the current study, we report the super-capacitive activity of the manufactured gCN and Cu_2_O-gCN, as a base substantial, in rapports of specific capacitance, energy and power densities, and electrochemical steadiness. Cyclic voltammetry (CV) and galvanostatic charge/discharge (GCD) analysis were initially done for explaining the electrochemical activity of the modified working GCE in a three-electrode scheme in a 0.5 mol·dm^−3^ KOH solution. Figure 6a (inset figure) presents the comparison of CV study of the gCN, Cu_2_O-gCN, and CuMnO_2_-gCN materials at a scan rate of 20 mV·s^−1^ within a voltage range of −0.25 to 0.3 V and the highest current density value of 0.41 and 0.10 A·g^−1^, correspondingly, was attained at 0.3 V. We optimized and chose this potential window for our experiment because, beyond this potential value, the current density was not stable for the repetitive number of cycles. To see the synergetic effect and to increase the conductivity of the Cu_2_O-gCN, we synthesized the CuMnO_2_-gCN. Figure 6a (main panel) demonstrates the voltammogram of the CuMnO_2_-gCN modified working electrode in 0.5 mol·dm^−3^ KOH at a potential sweep rate of 20 mV·s^−1^, and a considerable enhancement on the current performance, 3.21 A·g^−1^, was observed. The extraordinary growth in the connected CV area of the CuMnO_2_-gCN material in comparison with the Cu_2_O-gCN modified material is attributed to the combination of the high exterior area and electrochemical response of gCN. This suggests the higher charge storage aptitude and extensive development of the activity. The integral area should be distinguished, below which the CV curves of plain gCN incorporated GCE are lesser than those of the Cu_2_O-gCN and CuMnO_2_-gCN materials; exposing the insignificant influence of capacitance of the gCN supported material in entire specific capacitance (inset Figure 6a). The synergetic impact within the current density value of the CuMnO_2_-gCN incorporated electrode could be owing to the invention of the electronic and structural heterogeneity of the material. Now, the CuMnO_2_-gCN alloy has been adopted as the working substance for the remaining studies and constant enhancement of the current density conditions, as shown by the CV, has been marked with progressing scan rate from 20 to 400 mV·s^−1^, curves (a–h), sequentially (Figure 6b). The *C_S_* drops as the scan rate raises, which indicates the limited approachability of ions into the central section at a small-time range [56].

The outstanding capacitive activity of the synthesized electrode substances was also confirmed from GCD measurements (Figure 6c). The GCD measurements for the gCN, Cu_2_O-gCN, and CuMnO_2_-gCN composite incorporated electrodes, studied within the potential range from −0.25 V to 0.3 V, applying at a constant current density of 3.0 A·g^−1^ in 0.5 mol·dm^−3^ KOH. The *C_S_* values, gained from charge–discharge studies, of the corresponding composites were 0.8, 3.45, and 100.25 F·g^−1^, respectively, for the fixed current density of 3.0 A·g^−1^. Figure 6d exhibits the GCD cycles for the CuMnO_2_-gCN material incorporated electrodes, by applying a different current density value from 3.0 to 0.05 A·g^−1^ in 0.5 mol·dm^−3^ KOH. The *C_S_* data, found from charge–discharge studies, of the corresponding substantial were 100.25, 123.27, 165.58, 209.09, 225.32, 261.34, 332.09, 480.49, and 817.85 F·g^−1^ for the current density value of 3.0, 2.0, 1.0, 0.5, 0.35, 0.2, 0.1, 0.05, and 0.025 A·g^−1^, correspondingly. An extensive discharge period resembles a higher capacitance of the CuMnO_2_-gCN modified GCE, owing to a combing impact among gCN and CuMnO_2_ and an improved efficient surface area essentially aimed at ion exchange. To analyse the reliability and storage capacitance of the synthesized material, we used CuMnO_2_-gCN modified anode electrode in alkaline media at a fixed potential sweep rate and observed a considerable enhancement on the current performance. An extensive discharge period resembles a higher capacitance of the CuMnO_2_-gCN modified GCE, owing to a combing impact among gCN and CuMnO_2_ and an improved efficient surface area essentially aimed at ion exchange. From the GCD and CV studies, it was observed and confirmed that the durability and storage capability of our material has the potential for supercapacitor applications.

**Figure 7:**
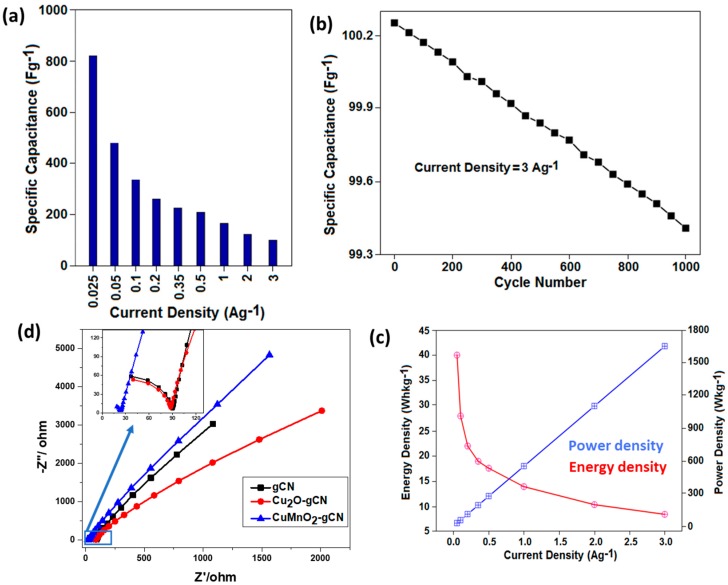
(**a**) Calculated specific capacitance of the CuMnO_2_-gCN sample. (**b**) Cycling stability performance of CuMnO_2_-gCN (the current density is 3 A·g^−1^). (**c**) The graphical relation between the energy density and power density as the function of current density for CuMnO_2_-gCN material, in the potential range of 3.0–0.1 V. (**d**) Nyquist plots of gCN, Cu_2_O-gCN, and CuMnO_2_-gCN in a frequency range of 3 MHz–10 Hz under an open circuit condition.

The graphical illustration, bar-chart (Figure 7a), denotes the current density vulnerability on the *C_S_* data. By varying the current density values from 0.025 to 3 A·g^−1^, the highest specific capacitance was attained while the experiment was carried out under the lowest input current density. For the energy storage application, the stability of the material is a significant restriction for the durability of the anode substance. We employed the GCD analysis to assess the stability of the CuMnO_2_-gCN anode material up to 1000 cycles. Figure 7b exhibits that CuMnO_2_-gCN anode material displayed higher rate steadiness around 91% of its initial capacitance after 1000 cycles, charging and discharging, and shows the high durability of the composite. The higher rate competence of the CuMnO_2_-gCN anode is accredited to the quicker diffusion extent of the ions, and the higher exterior area, including the increased electrochemical response.

Figure 7c displays the graphical illustration of the energy and power density of the material versus current density. With a reduction in the current density conditions, the energy density rises and the power density decreases. The nonlinear characteristics of the curve (energy versus current density) change with time, a variable factor that varies with the current value in comparison with the energy density. Electrochemical impedance spectroscopy (EIS) investigation was conducted to identify the electrical features of all the incorporated substances. The EIS study was carried out to determine the kinetic criterion of the gCN, Cu_2_O-gCN, and CuMnO_2_-gCN incorporated materials on a frequency scale from 3 MHz to 10 Hz at open-circuit voltages (Figure 7d). The Nyquist designs of the three-electrode elements are made of the slanted semicircles within the high-frequency range and vertical shapes during the low-frequency section, which represents frontier charge-transfer resistance (R_ct_) and dissemination resistance into the electroactive substance, sequentially [57,58]. The R_ct_ outcome is 86.9, 90.1, and 22.9 Ω aimed at the gCN, CuO_2_-gCN, and CuMnO_2_-gCN materials, sequentially. The CuMnO_2_-gCN electrode shows a more perpendicular line (50°) compared with the Cu_2_O-gCN (35°) and gCN electrode (25°); representing the lower dispersion resistance with more distinct capacitive performance. The operative assistance of gCN nanosheets as per the conductive outline within the CuMnO_2_-gCN nanocomposite is the looming reason for electrochemical activity development. The outcome specifies that the compound of CuMnO_2_-gCN structure has more ability to store the energy as compared with other base materials.

## 4. Conclusions

In this report, we reveal the synthesis of novel anode material for energy storage applications. We used a facile route to synthesize the copper–manganese alloy of diverse stoichiometric configurations that were supported on gCN nanosheets. The CuMnO_2_-gCN modified GCE reveals a better electrochemical response compared with the Cu_2_O-gCN electrode. The CuMnO_2_-gCN material exhibits a high specific capacitance (817.85 F·g^−1^ at 0.025 A·g^−1^) and great cycling stability (retention of 91% up to 1000 cycles). On the basis of the current results, the CuMnO_2_-gCN composite offers a piece of compelling evidence as a potential electrode material for use in progressive energy storage devices. SCs’ discovery in advance energy purposes vast energy capability including equipment by comparatively less time plus higher endurance. Recently, the integration of carbons including metal oxides/alloy to develop hybrid SCs can be described as a potential substitute for energy-related purposes.

## Supplementary Materials

The following are available online at https://www.mdpi.com/2079-4991/10/1/2/s1, Figure S1: (a,b) SEM images and (c) corresponding EDX elemental mapping of gCN; Figure S2: (a,b) SEM images and (c) corresponding EDX elemental mapping of Cu_2_O-gCN; Figure S3: TEM image of Cu_2_O-gCN (a), (b) STEM-HAADF images of Cu_2_O-gCN and corresponding EDX elemental mapping of the selected area, and (c) HRTEM image of Cu_2_O-gCN and corresponding FFT image of selected area in (c); Figure S4: XPS survey spectrum of CuMnO_2_-gCN nanocomposite.
