# Supplementary material for: Graphitic Carbon Nitride Doped Copper–Manganese Alloy as High–Performance Electrode Material in Supercapacitor for Energy Storage
Source: Nanomaterials (Basel). 2019 Dec 18;10(1):2. doi: 10.3390/nano10010002 (PMC7023178; doi:10.3390/nano10010002)
Supplement: Supplementary file 1 [file nanomaterials-10-00002-s001.pdf]

Graphitic Carbon Nitride Doped Copper-Manganese Alloy as High Performance Electrode Material for Supercapacitor Application

Samarjeet Singh Siwal [1,*], Qibo Zhang [1,2,*], Changbin Sun[1] and Vijay Kumar Thakur [3,4,*]

[1] Key Laboratory of Ionic Liquids Metallurgy, Faculty of Metallurgical and Energy Engineering, Kunming University of Science and Technology, Kunming 650093 P.R. China; changbin_sun@163.com
[2] State Key Laboratory of Complex Nonferrous Metal Resources Cleaning Utilization in Yunnan Province, Kunming 650093, P.R. China
[3] Enhanced Composites and Structures Center, School of Aerospace, Transport and Manufacturing, Cranfield University, Bedfordshire MK43 0AL, UK
[4] Department of Mechanical Engineering, School of Engineering, Shiv Nadar University, Uttar Pradesh 201314, India
* Correspondence: samarjeet6j1@gmail.com (S.S.S.); qibozhang@kust.edu.cn (Q.B.Z.); Vijay.Kumar@cranfield.ac.uk (V.K.T.)

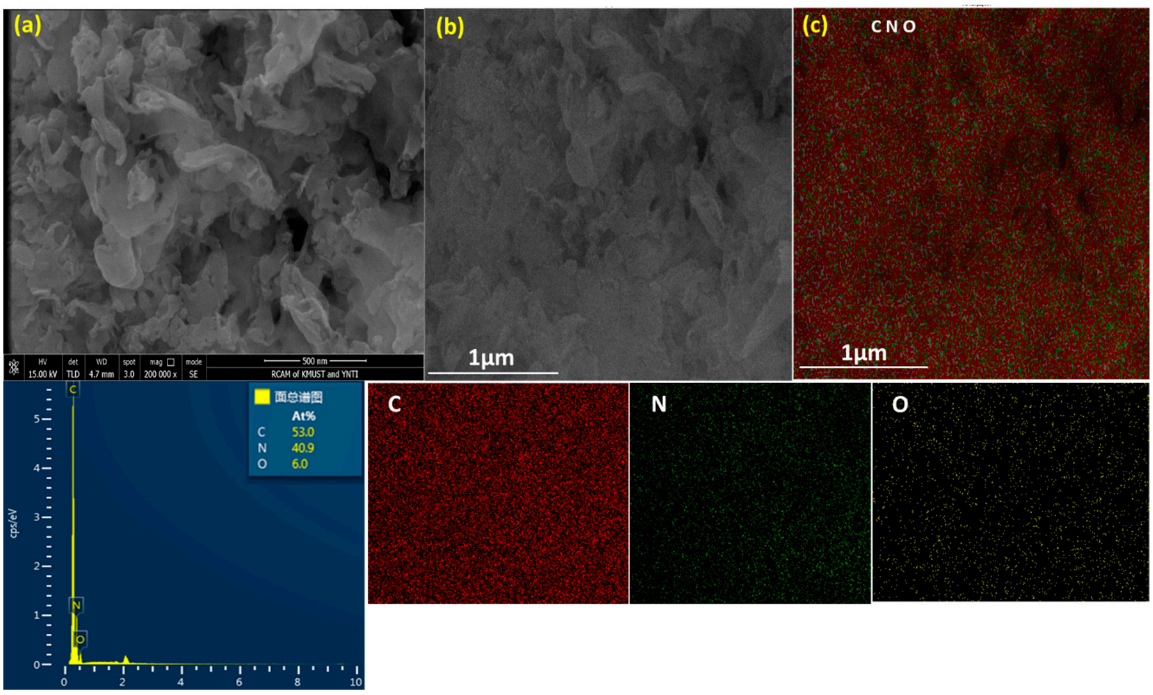

Figure S1. (a,b) SEM images and (c) corresponding EDX elemental mapping of gCN.

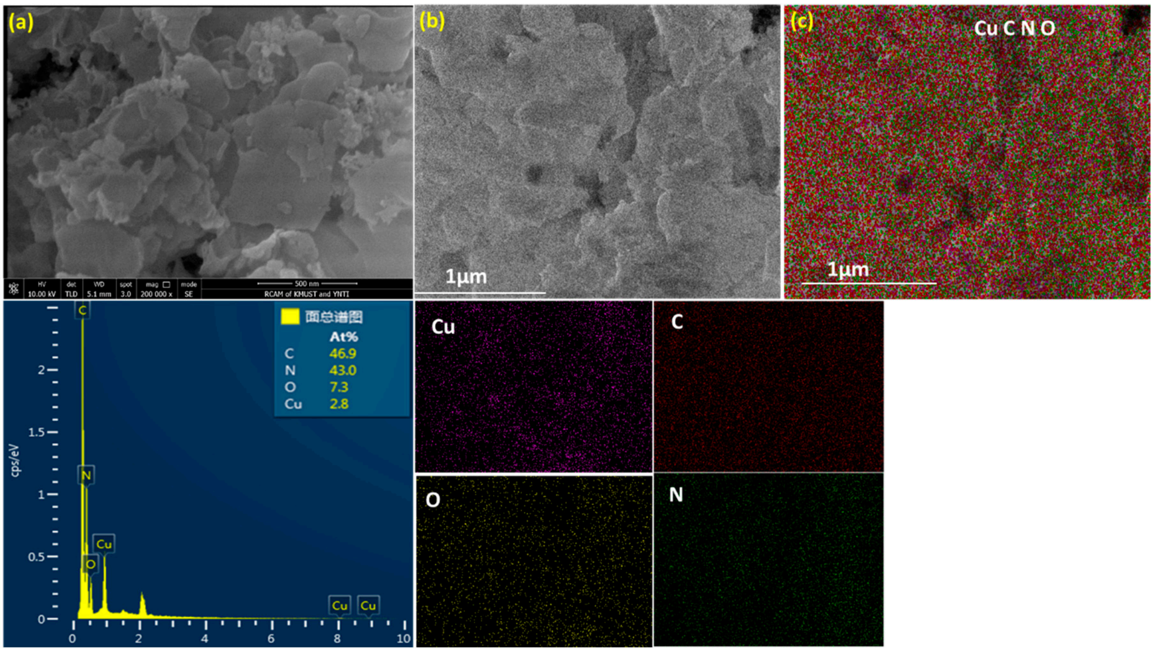

Figure S2. (a,b) SEM images and (c) corresponding EDX elemental mapping of Cu_2O-gCN.

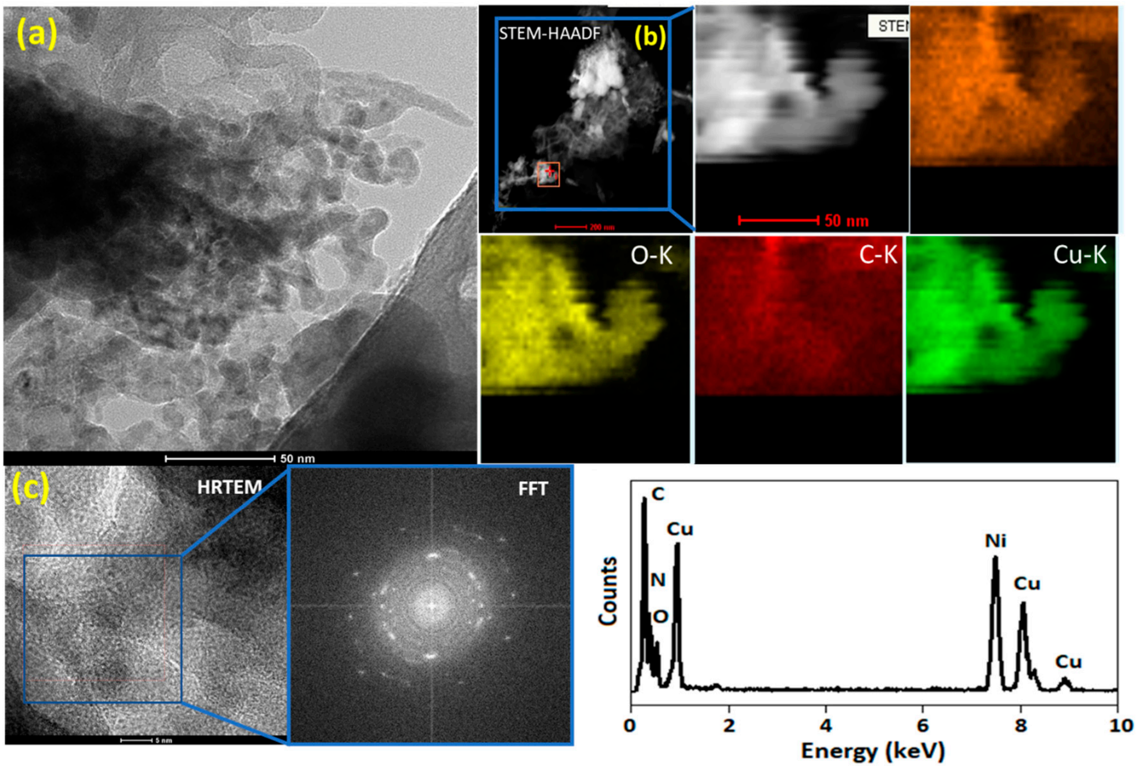

Figure S3. TEM image of Cu_2O-gCN (a), (b) STEM-HAADF images of Cu_2O-gCN and corresponding EDX elemental mapping of the selected area and (c) HRTEM image of Cu_2O-gCN and corresponding FFT image of selected area in (c).

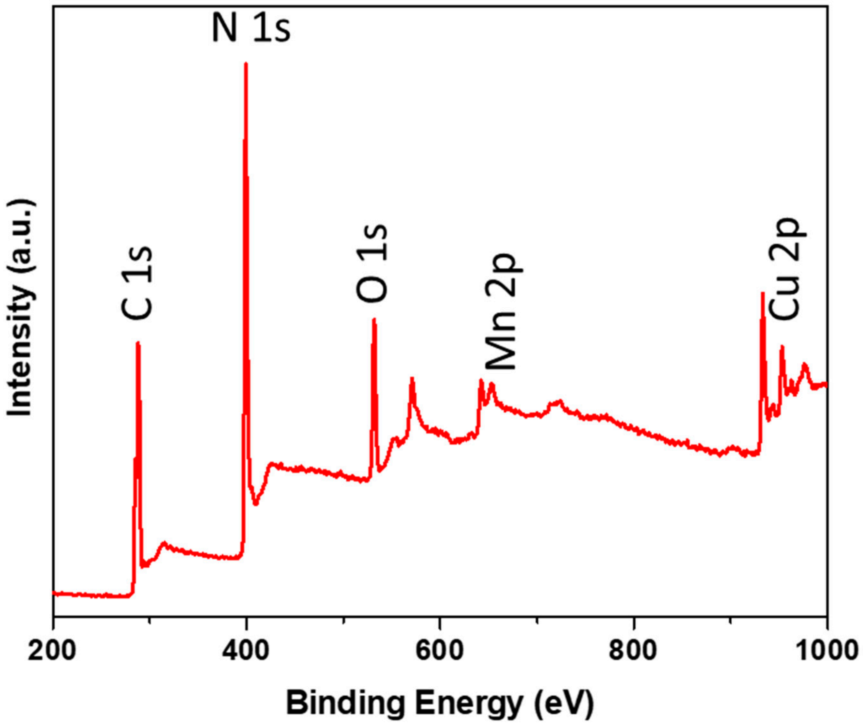

Figure S4. XPS survey spectrum of $CuMnO_2$-gCN nanocomposite.
